# Clinical efficacy of thymosin alpha 1 combined with multi-modality chemotherapy and its effects on immune function of patients with pulmonary tuberculosis complicated with diabetes

**DOI:** 10.12669/pjms.38.1.4419

**Published:** 2022

**Authors:** Li Wu, Pei-pei Luo, Yan-hong Tian, Lai-yin Chen, Yan-li Zhang

**Affiliations:** 1 Li Wu, Department of infection, Baoding People’s Hospital, Baoding 071000, Hebei, China; 2 Pei-pei Luo, Department of infection, Baoding People’s Hospital, Baoding 071000, Hebei, China; 3 Yan-hong Tian, Department of infection, Baoding People’s Hospital, Baoding 071000, Hebei, China; 4 Lai-yin Chen, Department of infection, Baoding People’s Hospital, Baoding 071000, Hebei, China; 5 Yan-li Zhang, Department of Infectious Diseases, North China University of Science and Technology Affiliated Hospital, Tangshan 063000, Hebei, China

**Keywords:** Pulmonary tuberculosis complicated with diabetes mellitus, Thymosin alpha 1, Immunoregulation, T cell, Sputum supernatant cytokine, Adverse reaction

## Abstract

**Objective:**

To observe the clinical efficacy of thymosin alpha 1 (Tα1) combined with multi-modality chemotherapy in patients with pulmonary tuberculosis (PTB) complicated with diabetes and discuss the effects of such combination therapy on lymphocyte subsets and sputum levels of cytokines.

**Methods:**

A total of 120 patients with PTB complicated with diabetes admitted to the Affiliated Hospital of North China University of Science and Technology from January 2017 to January 2018 were included in this study and randomly divided into an experimental group (Tα1 group, n=60) and a control group (n=60). Clinical efficacy and adverse drug reactions were observed and compared between the two groups. Blood samples were collected for lymphocyte (NK cell and T cell subsets) levels by flow cytometry, and sputum samples were collected for cytokine (IL-2, IFN-γ, IL-4 and TNF-α) levels by ELISA.

**Results:**

Two groups showed no statistically significant difference in sputum culture-negative conversion rate, chest lesion absorption rate, and cavity closure rate (P>0.05) after 6 months of treatment. However, after 12 months, the sputum culture-negative conversion rate, chest lesion absorption rate, and cavity closure rate in the Tα1 group increased compared with the control group, and the differences were statistically significant (P<0.05). There was a significant increase in CD3+, CD4+, NK-cells lymphocytes after six months in the Tα1 group than in the control group, whereas the CD8+, Th17, Treg lymphocytes in the Tα1 group were substantially lower than in the control group, with the differences showing statistical significance (P<0.05, respectively). After six months of treatment, the sputum supernatant levels of interleukin-4 (IL-4) and tumor necrosis factor α (TNF-α) in the Tα1 group were lower than in the control group, whereas the sputum supernatant levels of interleukin-2 (IL-2) and interferon gamma (IFN-γ) in the Tα1 group were higher than in the control group, and the differences were statistically significant (P<0.05, respectively). There was no statistically significant difference in the incidence of adverse reactions between the two groups (P>0.05).

**Conclusion:**

Tα1 combined with multi-modality chemotherapy has a visible curative effect on PTB patients with diabetes as it can regulate immune function and reduce the levels of inflammatory cytokines. As a safe combination therapy, it seems promising for further use in clinical practice.

## INTRODUCTION

As pointed out by the WHO in the Global Tuberculosis (TB) Report 2012, China is heavily burdened with TB.^1^ Pulmonary tuberculosis (PTB) is a chronic infectious respiratory disease caused by the bacterium *Mycobacterium tuberculosis* (*M. tb*) and primarily affecting the lungs. If an individual does not timely receive effective treatment, PTB can lead to cavity expansion and focal fibrosis due to adverse drug reactions, bacterial variation, hepatitis B virus (HBV), human immunodeficiency virus (HIV), or diabetes.^2^ Clinically, medication remains the mainstay of treatment for patients with PTB, in which case, the curative effect, and clinical prognosis are strongly affected by the relatively long course of treatment and high drug tolerance.^3^,^4^ Diabetes is a contributory factor for impairment of the immune mechanism, hypofunction of the immune system, and an increased risk of TB infection.^5^ In addition to abnormal carbohydrate metabolism, diabetes is also associated with lipid and protein metabolism at an accelerated speed. This creates a favorable environment for the growth of *M. tb* and reduces the curative effect of anti-TB treatment for PTB patients with diabetes.^6^ Thymosins are small molecular polypeptide secreted by thymus tissue, which modulate both innate and adaptive immunity, can reverse T cell failure and restore immune reconstruction.^7^ Thymosin alpha 1 (Tα1) and thymopentin (TP-5) are two thymus-derived immunomodulatory agents. Immunomodulatory role of thymosin is established not only in microbial infections but also in malignancy, pancreatic lesions, diabetes, immunodeficiencies, and vaccine efficacy etc.^8^

In this study, PTB patients with diabetes were treated with Tα1 injections in combination with conventional multi-modality chemotherapy to modulate the immune system, without mitigating the anti-TB effect.

## METHODS

### Ethical approval:

The study was approved by the Institutional Ethics Committee of North China University of Science and Technology Affiliated Hospital on June 10, 2018 (No.: 2041ZF147), and written informed consent was obtained from all participants.

### Clinical data:

In this retrospective study 120 patients who were admitted to North China University of Science and Technology Affiliated Hospital between January 2017 and January 2018 due to PTB complicated with diabetes were included according to the random sampling principle, and divided into two groups by random number table method, namely the Tα1 group (n=60) and the control group (n=60).

### Inclusion criteria:

A patient was considered eligible for the clinical trial if he/she:

1. Was confirmed to have PTB and diabetes according to the WS 288-2008 Diagnostic criteria for pulmonary tuberculosis^9^ and the 2014 American Diabetes Association (ADA) diabetes guidelines;^10^
2. Hhad not received any treatment that might affect the immune function within the past year.

### Exclusion criteria:

if the patient:

1. Was diagnosed with HIV infection, immune deficiency, or allergic disorders;
2. Presented with serious organ dysfunction of heart, liver, or kidneys;
3. Had AIDS or cancer;
4. Was pregnant or breastfeeding;
5. Have used glucocorticoids (GCs) in the last three months. Age, sex, course of disease, cavity proportion, grade of acid-fast bacilli in sputum smear and location of lesion were comparable between the two groups. (shown in Table-I)

**Table I:** Pre-treatment clinical data.

Group	Number of Patients	Sex		Age	Course of Disease	Number of Cavities	Involved Lung Field(s)		Sputum AFB Grades			

	n	Male	Female	(years)	(months)		1-4	≥4	1+	2+	3+	4+
Tα1	60	36	24	46.68±9.44	6.85±2.78	56	26	34	10	24	20	6
Control	60	40	20	47.53±9.62	6.77±2.12	54	22	38	12	18	22	8
X^2^/t		0.287		0.345	0.125	0.000	0.281		0.111	0.660	0.071	0.001
P		>0.05		>0.05	>0.05	>0.05	>0.05		>0.05	>0.05	>0.05	>0.05

### Therapeutic regimens:

1. The multi-modality anti-TB chemotherapy 2HRZE/4HR (H: 0.3 g isoniazid, R: 0.6 g rifampicin, Z: 1.5 g pyrazinamide, and E: 0.75 g ethambutol) was initiated and administered on alternate days, with the intensive phase containing isoniazid, rifampicin, pyrazinamide and ethambutol, and the continuation phase with isoniazid plus rifampicin. In case sputum smears did not convert after two months of treatment, the intensive phase should be extended for another month. If necessary, chemotherapy could be extended up to nine months according to CT findings and sputum smear results. In addition to chemotherapy, the Tα1 group also received subcutaneous injections of 120 mg/d Tα1 for 12 weeks.
2. NovoMix 30 was injected subcutaneously 10 minutes before breakfast and dinner every day. The recommended starting dose was 0.2-0.3 IU/kg/d, which should be adjusted according to the results of blood glucose monitoring. For patients who responded poorly to NovoMix 30, 4 mg/d rosiglitazone was administered to improve insulin sensitivity. Target blood glucose levels are as follows: fasting blood glucose (FBG) <7.0 mmol/L, 2h postprandial blood sugar (2hPBG) <10.0 mmol/L, and bedtime blood glucose (BBG) <9.0 mmol/L.

### Outcome measures:

***Response evaluation criteria:*** Sputum smear microscopy and plain radiography of the chest were performed monthly during the treatment course. Cavity closure, lesion absorption, and sputum culture-negative conversion before and after treatment (6 and 12 months after treatment) were recorded: a. sputum culture-negative conversion: sputum culture converted to negative and was not retested positive following two months of treatment; b. lesion absorption: radiography of the chest indicated a reduction of lesion area; c. cavity closure: radiography of the chest revealed closure, obstructive closure, or shrinkage of cavities.

***Measurement of lymphocytes (T cell subsets and NK cells):*** Before treatment and six months after treatment, 5ml of fasting anticoagulant whole blood was collected. Peripheral blood mononuclear cells were immediately sent to the laboratory for isolation and stored at 4°C. Within 3 days, CD3+, CD4+, CD8+, CD4+/CD8+, Th17, Treg, Th17/Treg, and NK (natural killer) cells were detected by Cyto-FLEX flow cytometry. All samples were stained according to the procedure of CD45/ CD8/CD4/ CD3 lymphocyte test kit, then analyzed on the machine. In the operating system, template loops were set to circle total lymphocytes, T cells (CD3+/CD4+/CD8+), Th17 cells (CD3+CD4+), Treg cells (CD4+CD25+), and NK cells (CD3-CD16+CD56+) respectively. At least 5000 cells under the lymphocyte subset were obtained. The relative percentage of each T lymphocyte subset in the total lymphocytes was obtained in the software.

***Sputum cytokines:*** Deep sputum was collected before treatment and six months after treatment. After a night of fasting, patients were asked to gargle with water and sputum aspiration was induced in a negative pressure chamber. After sputum collection, the sputum was stored at 4°C and immediately transported to the laboratory for treatment within two hour. The sputum was diluted and centrifuged to obtain the supernatant and stored at -70°C until cytokine levels were evaluated. Kits from Medgenix (Biosource International, Camarillo, Calif.) were used to determine gamma IFN-γ and TNF-α immunoreactivities. While kits from Peprotech (The United States) were used to detect IL2 and IL4.

***Adverse reactions/drug toxicity:*** Adverse reactions such as transaminase (AST) elevation, uric acid (UA) elevation, nausea and vomiting, rash, drowsiness, and hypoglycemia were recorded during the treatment course.

### Statistical analysis:

Statistical analysis was performed using SPSS 22.0 software. Measurement data were expressed in the form of “mean ± standard deviation (x±s)”, and intergroup comparison was examined with the independent-samples t-test. Quantitative data were represented by percentages (%), and intergroup comparison was examined with the χ2 test. The significance level α was set at 0.05.

## RESULTS

After six months of treatment, the two groups showed no statistically significant differences in sputum culture-negative conversion rate, chest lesion absorption rate, and cavity closure rate (P>0.05); following 12 months of treatment, the sputum culture-negative conversion rate, chest lesion absorption rate, and cavity closure rate in the Tα1 group increased compared with the control group, and the differences were statistically significant (P<0.05) (Table-II).

**Table II:** Comparison of clinical efficacy [n/%].

Group	Number of Patients	Sputum Culture-Negative Conversion Rate		Chest Lesion Absorption Rate		Cavity Closure Rate	

	n	6 months post-treatment	12 months post-treatment	6 months post-treatment	12 months post-treatment	6 months post-treatment	12 months post-treatment
Tα1	60	32 (53.33)	44 (73.33)	36 (60.00)	48 (80.00)	30 (50.00)	46 (76.67)
Control	60	18 (30.00)	26 (43.33)	22 (36.67)	30 (50.00)	16 (26.67)	28 (30.00)
χ2		3.360	5.554	3.270	5.934	3.455	5.711
P		>0.05	<0.05	>0.05	<0.05	>0.05	<0.05

### Comparison of NK and T-cell subsets:

After six months of treatment, the CD3+, CD4+, CD4+/CD8+, and NK cells in the Tα1 group were remarkably higher than in the control group, whereas the CD8+, Th17, Treg, and Th17/Treg cells in the Tα1 group were substantially lower than in the control group, and the differences were statistically significant (P<0.05, respectively) (Table-III).

**Table III:** Comparison of T-cell subsets (x±s) after 6 months of treatment.

Group	CD3+ (%)		CD4+ (%)		CD8+ (%)		CD4+/CD8+ (%)	

	Pre-treatment	Post-treatment	Pre-treatment	Post-treatment	Pre-treatment	Post-treatment	Pre-treatment	Post-treatment
Tα1	51.01±2.00	68.81±2.51	31.33±4.47	41.68±4.62	32.26±4.74	26.57±6.83	1.05±0.15	1.79±0.71
Control	51.40±1.81	56.62±2.30	31.58±4.22	32.48±2.61	32.10±3.20	32.16±4.69	1.08±0.32	1.12±0.68
t	0.256	0.963	0.256	7.553	0.176	4.154	0.531	4.282
P	>0.05	<0.001	>0.05	<0.001	>0.05	<0.001	>0.05	<0.001

**Group**	**Th17 (%)**		**Treg (%)**		**Th17/Treg (%)**		**NK cells (%)**	

	**Pre-treatment**	**Post-treatment**	**Pre-treatment**	**Post-treatment**	**Pre-treatment**	**Post-treatment**	**Pre-treatment**	**Post-treatment**

Tα1	2.41±0.74	2.17±1.06	4.48±0.72	4.29±1.23	0.52±0.15	0.51±0.16	13.31±1.52	19.05±3.40
Control	2.57±0.71	3.84±0.92	4.46±0.68	5.48±1.73	0.51±0.16	0.61±0.20	13.28±1.47	15.52±2.89
t	1.209	5.680	0.981	9.068	0.895	5.242	0.380	3.446
P	>0.05	<0.05	>0.05	<0.05	>0.05	<0.05	>0.05	<0.01

### Comparison of sputum supernatant levels of cytokines:

After six months of treatment, the sputum supernatant levels of IL-4 and TNF-α in the Tα1 group were lower than in the control group, whereas the sputum supernatant levels of IL-2 and IFN-γ in the Tα1 group were higher than in the control group, and the differences were statistically significant (P<0.05, respectively) (Table-IV).

**Table IV:** Comparison of sputum supernatant levels of cytokines (x±s) after 6 months of treatment.

Group	n	IL-2 (pg/L)		IL-4 (ng/L)		TNF-α (ng/L)		IFN-γ (ng/L)	

		Pre-treatment	Post-treatment	Pre-treatment	Post-treatment	Pre-treatment	Post-treatment	Pre-treatment	Post-treatment
Tα1	60	163.4±34.7	108.0±24.1	189.85±122.64	64.22±42.27	552.12±261.23	123.45±102.56	6.03±2.78	22.26±4.19
Control	60	158.3±31.8	75.1±19.1	190.87±124.65	127.83±135.67	546.78±256.89	241.23±189.9	5.97±3.11	12.45±4.55
t		0.702	6.771	0.045	3.438	0.112	4.190	0.110	12.182
P		>0.05	<0.001	>0.05	<0.001	>0.05	<0.001	>0.05	<0.001

### Adverse reactions/drug toxicity:

There was no statistically significant difference in the incidence rates of AST elevation, UA elevation, nausea and vomiting, rash, drowsiness, and hypoglycemia between the two groups (P>0.05) (Table-V).

**Table V:** Comparison of Adverse reactions/drug toxicity.

Group	Number of Patients	AST Elevation	UA Elevation	Nausea and Vomiting	Rash	Drowsiness	Hypoglycemia	Total
Tα1	60	2 (3.33)	2 (3.33)	2 (3.33)	4 (6.66)	0 (0)	2 (3.33)	12 (20.0)
Control	60	2 (3.33)	2 (3.33)	2 (2.38)	0 (0)	0 (0)	2 (3.33)	8 (13.3)
χ2								0.480
P								>0.05

## DISCUSSION

Diabetes is a chronic wasting disease characterized by disorders of carbohydrate and protein metabolism, inhibition of antibody synthesis, and defects in immune function.^11^ *M. tb* can directly downregulate the expression of Th1 cells, and TB can lead to immune dysfunction.^12^,^13^ Diabetes is an important risk factor for secondary PTB, and TB poisoning symptoms, and anti-TB drugs can interfere with carbohydrate metabolism and lead to exacerbations of immune disorders.^14^,^15^ Poor blood sugar control can compromise anti-TB treatment. Considering that abnormal pathogenic immune response cannot be directly corrected by conventional anti-TB therapy, the key to a higher success rate of anti-TB treatment lies in improving cellular immunity.^16^ Thymosins are important immunomodulatory agents extensively used for adjuvant treatment of chronic infectious diseases.^17^,^18^ Thymosins, Tα1 restore and improve immune function significantly.^19^

In this study, it was found that the Tα1 group outperformed the control group in sputum culture-negative conversion, lesion absorption, and cavity closure, indicating desirable clinical efficacy in treating PTB complicated with diabetes with Tα1 and multi-modality chemotherapy. This is because thymosins stimulate T-cell production by the bone marrow to improve immune function, thereby eliminating dormant or persistent bacteria in combination with anti-TB drugs. This study also revealed that the CD3+, CD4+, CD4+/CD8+ T cells, and NK-cell values in the Tα1 group were significantly higher than in the control group, whereas the CD8+, Th17, Treg, and Th17/Treg values were significantly lower than in the control group. It has been reported that Treg cells are involved in immune disorders during sepsis, and they lead to Th2 immune response, while Tα1 can down-regulate the percentage of Treg and reduce inflammatory damage.^20^ Our findings are similar. Other studies have found that Tα1 can up-regulate Treg levels in peripheral blood of patients with gastric cancer.^21^ We believe that the decrease of Treg and Th17 subgroups in the experimental group in this study may be related to the complex immune response involved in tuberculosis combined with diabetes and the differences in individual immune status. In the development and progression of PTB, it is significant to inhibit the expression of Th1 cells and increase the expression of Th2 cells.^22^ Thymosins promote differentiation and proliferation of T cells and support Th1/Th2 balance by regulating CD3+, CD4+, and CD8+ T cells, as well as the CD4+/CD8+ and Th17/Treg ratios.^23^ In addition, thymosins can improve the cytolytic and killing abilities of NK cells and enhance the phagocytic function of macrophages, thereby killing *M. tb*.^24^ IFN-γ is a multifunctional immunomodulator and cytokine produced by T cells upon stimulation by specific antigens. IL-2 and IFN-γ support Th1 cell proliferation and antagonize the effects of IL-4 and TNF-α.^25^ All this conforms with the findings of this study: the Tα1 group had lower sputum supernatant levels of IL-4 and TNF-α but higher sputum supernatant levels of IL-2 and IFN-γ compared with the control group. Previous studies have also shown that Tα1 can reduce liver inflammation and reduce hepatocyte apoptosis by down-regulating TNF-α and up-regulating IL-10.^26^ Adverse drug reactions occurring in the patients of the two groups during treatment mainly include an increase in AST and UA, nausea and vomiting, rash, and hypoglycemia. These symptoms are caused by anti-TB and hypoglycemic drugs and tend to be mild and reversible. No adverse reactions are produced by the Tα1 therapy.

### Limitations of this study

There are still some shortcomings in this study. The number of subjects included in this study is limited, so the conclusions drawn may not be very convincing. In addition, further studies with large-scale standard treatment are needed to verify the curative effect, safety, optimum dose, and duration of thymosin treatment.

## CONCLUSION

Tα1 combined with multi-modality chemotherapy has a visible curative effect on PTB patients with diabetes as it can regulate immune function and reduce the levels of inflammatory cytokines. As a safe combination therapy, it seems promising for further use in clinical practice.

### Authors’ Contributions:

**LW and YZ** designed this study, prepared this manuscript and are responsible, accountable for the accuracy or integrity of the work.

**PL and YT** collected and analyzed clinical data.

**LC** significantly revised this manuscript.
